# The efficacy of neoadjuvant *EGFR*-TKI therapy combined with radical surgery for stage IIIB lung adenocarcinoma harboring *EGFR* mutations: A retrospective analysis based on single center

**DOI:** 10.3389/fonc.2023.1034897

**Published:** 2023-01-26

**Authors:** Yicheng Xiong, Dongliang Bian, Zhida Huang, Huansha Yu, Jie Huang, Peng Zhang, Wenxin He, Hongcheng Liu

**Affiliations:** ^1^ Department of Thoracic Surgery, Shanghai Pulmonary Hospital, Tongji University School of Medicine, Shanghai, China; ^2^ Medical Graduate School, Nanchang University, Nanchang, China; ^3^ Department of Animal Experiment Center, Shanghai Pulmonary Hospital, Tongji University School of Medicine, Shanghai, China; ^4^ Department of Clinical Research Center, Shanghai Pulmonary Hospital, Tongji University School of Medicine, Shanghai, China

**Keywords:** non-small cell lung cancer, adenocarcinoma, neoadjuvant targeted therapy, epidermal growth factor receptor, stage IIIB

## Abstract

**Background:**

Epidermal growth factor receptor tyrosine kinase inhibitors (*EGFR*-TKIs) could provide survival benefits for locally advanced *EGFR*-mutant (*EGFR*m) non-small cell lung cancer (NSCLC). However, the role of radical surgery for *EGFR*-TKI treated stage IIIB *EGFR*m NSCLC remains controversial. This study attempted to assess the feasibility of neoadjuvant *EGFR*-TKI followed by radical surgery for stage IIIB *EGFR*m NSCLC.

**Patients and Methods:**

Between 2013 and 2020, *EGFR*m lung adenocarcinoma (LUAD) patients in clinical stage IIIB undergoing neoadjuvant *EGFR*-TKI followed by surgery (T-S-Arm) and *EGFR*-TKI alone (T-Arm) were reviewed retrospectively in Shanghai Pulmonary Hospital (SPH). The chi-square test, Student’s *t*-test or Fisher’s exact test was performed for analysis of baseline characteristics. Progression-free survival (PFS) was estimated using the Kaplan-Meier analysis. Multivariate Cox regression analysis was used to identify independent predictors of progression.

**Results:**

A total of 43 patients were divided into T-S-Arm (n = 21) and T-Arm (n = 22). Patients were well-balanced between the two arms. The majority of patients were female (n = 25, 58.1%), non-smokers (n = 35, 81.4%), first-generation of *EGFR*-TKI treatment (n = 39, 90.7%), and exon 19 deletions (19-DEL) (n = 26, 60.5%). The median diagnostic age was 63.0 years [interquartile range (IQR), 54.0-67.5 years). At the cut-off date with June 30th 2022, median follow-up time was 28 months (IQR, 20-39 months). Neoadjuvant *EGFR*-TKI treatment followed by radical surgery could significantly improve the median PFS compared with patients underwent *EGFR*-TKI alone (23.0 months vs 14.5 months, *P* = 0.002). Multivariate Cox regression analysis demonstrated that radical surgery (T-S-Arm vs. T-Arm, HR: 0.406; 95% CI: 0.207-0.793, *P* = 0.027) was the only independent predictor for disease progression. The stratified analysis demonstrated patients with N2 disease could benefit from radical surgery (HR, 0.258; 95% CI, 0.107-0.618), especially for patients harboring L858R mutation (HR, 0.188; 95% CI, 0.059-0.604).

**Conclusions:**

For stage IIIB *EGFR*m NSCLC patients, the prognosis might be improved by neoadjuvant *EGFR*-TKI followed by radical surgery versus *EGFR*-TKI alone, especially for those with N2 disease and harboring L858R mutation.

## Introduction

Non-small cell lung cancer (NSCLC) represents approximately 85% of lung cancer worldwide (1), and 22% of NSCLC patients were diagnosed with locally advanced (stage III) disease (2). NSCLC in stage III is divided into IIIA to C (3). NSCLC patients with stage IIIB disease, considering unresectable disease, have limited benefits from surgery followed by adjuvant treatment (4). In 2014, the multicenter phase II study (TAX-AT 1.20 trial) demonstrated neoadjuvant chemotherapy with docetacxel/cisplatin followed by complete resection could improve prognosis of NSCLC patients in stage II, IIIA and IIIB (5). ASCO guideline has recommended that patients with unresectable stage III disease may be offered induction therapy followed by complete resection (6).

The percentage of Asian NSCLC patients with epidermal growth factor receptor (*EGFR*) mutations is 30%, and the most common mutations are deletions in exon 19 (19-DEL) and the exon 21 codon p.Leu858Arg point (L858R) mutation (7). In patients with *EGFR* mutations, *EGFR*-TKI therapy has shown good efficacy and well-tolerance compared with chemotherapy (8). In recent years, studies showed neoadjuvant *EGFR*-TKI therapy could downstage the tumor and improve the rate of radical surgery in patients with locally advanced NSCLC. Xiong et al. demonstrated that neoadjuvant erlotinib therapy could improve the rate of radical surgery (13/19, 69.4%) of *EGFR*m NSCLC patients in stage IIIA-N2, achieving a median progression-free survival (PFS) of 12.1 months (95% CI, 9.4-31.8) (9). Zhang et al. demonstrated the feasibility of neoadjuvant gefitinib therapy followed by radical surgery for *EGFR*m NSCLC patients in stage II-IIIA [disease-free survival (DFS), 33.5 months, 95% CI, 19.7-47.3], and patients with major pathologic response (MPR, proportion of patients with no more than 10% residual viable tumor cells) had a better prognosis (DFS, *P* = 0.019) (10).

The safety and efficacy of neoadjuvant *EGFR*-TKI therapy for locally advanced *EGFR*m NSCLC have been confirmed by clinical trials above mentioned. However, no clinical trial focused on the efficacy of neoadjuvant targeted therapy combined with radical surgery for stage IIIB NSCLC harboring *EGFR* mutations. The efficacy of complete resection based on tumor downstaging after treated with neoadjuvant *EGFR*-TKI therapy remains controversial for *EGFR*m NSCLC patients diagnosed as stage IIIB disease. In this study, we attempted to assess the clinical efficacy of radical surgery after induction targeted therapy for *EGFR*m lung adenocarcinoma (LUAD) patients with stage IIIB disease.

## Methods

### Patient selection

We retrospectively included the NSCLC patients between January 2013 and December 2020 in Shanghai Pulmonary Hospital (SPH). The approval of the study was granted by Ethical Committee of Shanghai Pulmonary Hospital, and informed consent was obtained by all patients. The inclusion criteria were as followed: (I) patients harboring L858R mutation or 19-DEL confirmed by molecular biological detection; (II) patients diagnosed as NSCLC with clinical stage IIIB disease; (III) the diagnostic age of patients elder than 18 years; (IV) Eastern Cooperative Oncology Group (ECOG) performance status was 0 to 1; (V) radical surgery could not be completed at the time of diagnosis. The exclusion criteria were as followed: (I) history of cancer within 5 years; (II) primary resistance to *EGFR*-TKIs; (III) other systemic neoadjuvant antitumor therapy before preoperative evaluation. These patients underwent neoadjuvant *EGFR*-TKI followed by surgery (T-S-Arm) and *EGFR*-TKI alone (T-Arm) respectively. The pathological diagnosis of NSCLC was established on the basis of needle biopsy or endobronchial ultrasound (EBUS). All patients were pathologically diagnosed as LUAD. The type of *EGFR* mutations was confirmed by amplification refractory mutation system-polymerase chain reaction (ARMS-PCR). The NSCLC stage of all patients was evaluated by pathological detection and/or standardized uptake value (SUV) in positron emission tomography/CT (PET/CT). The stage of the primary tumor (T), lymph node (N), and metastasis (M) were evaluated based on the American Joint Committee on Cancer (AJCC) 8th edition TNM staging system for NSCLC (3). Patients with pre-induction N3 disease in T-S-Arm had downstaging and their N3 lymph nodes were negative after *EGFR*-TKI therapy, which was confirmed by PET/CT and ultrasound-guided fine-needle aspiration. In T-S-Arm, all patients should be performed surgery within 3 weeks after *EGFR*-TKI discontinuation. For patients received the first-generation *EGFR*-TKI, at least three days interval from *EGFR*-TKI discontinuation to surgery, and for patients received the second-generation *EGFR*-TKI treatment, the interval should be extended to 7 days. All patients in T-S-Arm were strongly required to undergo complete resection for LUAD after neoadjuvant treatment.

### Efficacy assessment

To evaluate the treatment response after *EGFR*-TKI treatment, chest CT images of all patients were reviewed and evaluated by radiologists based on the Response Evaluation Criteria in Solid Tumors (RECIST, version 1.1) (11). The tumor responses were classified as progressive disease (PD, ≥20% increased in size or the occurrence of now lesions), stable disease (SD, change in size between -30% to +20%), partial response (PR, ≥30% reduced in size) and complete remission (CR, no resident lesion). Chest CT and EBUS were performed to evaluate the lymph node response after *EGFR*-TKI treatment and to assess the surgical feasibility. Brain magnetic resonance imaging (MRI) and PET/CT or bone emission computed tomography (ECT) scan were performed to confirm the absence of distant metastasis.

### Follow-up strategy

Follow up was conducted by outpatient visits or telephone calls. For postoperative patients, physical examinations and chest CT were performed every 3 months for the first year, every 6 months for 2 to 5 years, and annually from then on. Brain MRI, ultrasonography of abdominal and bone ECT were performed annually or physicians considered necessary. For patients without surgery, physical examinations and chest CT were performed every 2 months. The cutoff date was June 30th 2022. PFS was defined as the interval of time from the beginning of treatment to the first progression or last follow-up. OS was defined as the interval of time from the beginning of treatment to death or last follow-up. Data was censored at the last follow-up for patients without recurrence or death.

### Statistical analysis

The statistical analysis used R software (R v.4.1.3). The chi-square test or fisher’s exact test and Student’s *t*-test were used for comparing the differences of categorical and continuous variables between T-S-Arm and T-Arm. PFS was analyzed by Kaplan-Meier method and was compared using the log-rank test. The stratified analyses of PFS were performed with the Cox proportional hazards model according to clinical characteristics. Multivariate Cox regression was used to evaluate independent survival predictors of progression, and factors with *P* < 0.1 from the univariate Cox regression was included in the multivariate Cox regression. *P* < 0.05 indicated statistical significance.

## Results

### Patient characteristics

**Figure 1:**
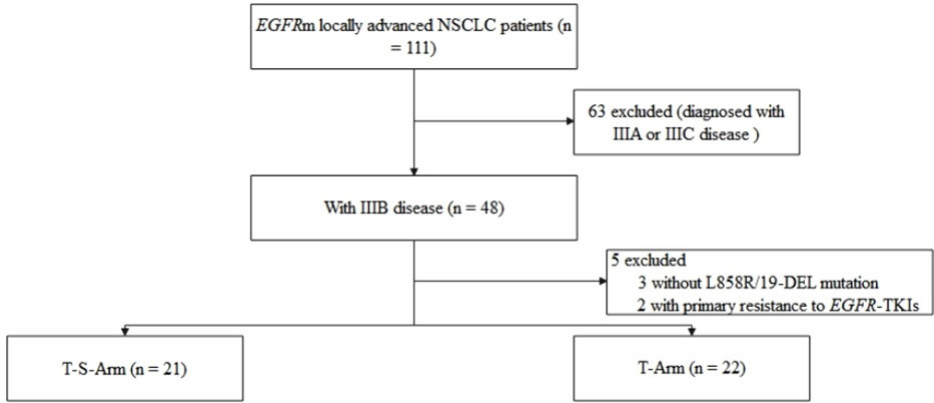
Study flow diagram. *EGFR*, epidermal growth factor receptor; NSCLC, non-small cell lung cancer; *EGFR*-TKI, epidermal growth factor receptor tyrosine kinase inhibitor.

A total of 43 *EGFR*m LUAD patients with clinical stage IIIB were included in this study retrospectively (21 in T-S-Arm and 22 in T-Arm respectively) (**Figure 1**). The characteristics of patients were summarized in **Table 1**. The median age was 63 years [interquartile range (IQR), 54.0-67.5]. The majority of patients were female (n = 25, 58.1%), non-smokers (n = 35, 81.4%), the first-generation *EGFR*-TKIs treated (n = 39, 90.7%), and harboring 19-DEL (n = 26, 60.5%). No significant difference was observed in the distribution of age, gender, smoking history and mutation subtypes between T-S-Arm and T-Arm. While, compared with patients in T-Arm, T-S-Arm patients had larger target lesions (57.0mm vs 37.9mm, *P* = 0.007). There were two (9.5%) patients with single N2 disease in T-S-Arm. There were 15 (15/21, 71.4%) patients in T-S-Arm receiving neoadjuvant therapy for up to 2 months (range, 1-2 months), and 6 (6/21, 28.6%) for more than 2 months (range, 3-6 months).

**Table 1:** Baseline information of patients receiving *EGFR*-TKIs.

	Total (N=43)	T-S-Arm (N=21)	T-Arm (N=22)	
**Median age, years (IQR)**	63 [54.0, 67.5]	61.0 [53.0, 67.0]	64.0 [59.0, 68.5]	0.225
Gender				0.223
Male	18 (41.9%)	11 (52.4%)	7 (31.8%)	
Female	25 (58.1%)	10 (47.6%)	15 (68.2%)	
Smoking history				0.457
Current or ever	8 (18.6%)	5 (23.8%)	3 (13.6%)	
Never	35 (81.4%)	16 (76.2%)	19 (86.4%)	
The Generation of TKI				-
I	39 (90.7%)	17 (81.0%)	22 (100%)	
II	4 (9.3%)	4 (19.0%)	0 (0%)	
Mutation Subtype				0.124
L858R	17 (39.5%)	11 (52.4%)	6 (27.3%)	
19-DEL	26 (60.5%)	10 (47.6%)	16 (72.7%)	
** Mean target lesions, mm (IQR)**	46.5 [30.3-60.5]	57.0 [36.0-72.0]	37.9 [22.0-50.0]	0.007
T stage				0.588
T1	8 (18.6%)	3 (14.3%)	5 (22.7%)	
T2	13 (30.2%)	5 (23.8%)	8 (36.4%)	
T3	9 (20.9%)	5 (23.8%)	4 (18.2%)	
T4	13 (30.2%)	8 (38.1%)	5 (22.7%)	
N stage				0.227
N2	22 (51.2%)	13 (61.9%)	9 (40.9%)	
N3	21 (48.8%)	8 (38.1%)	13 (59.1%)	
LN station				0.277
Single station N2	2 (4.7%)	2 (9.5%)	0 (0%)	
Multiple station N2	21 (48.8%)	11 (52.4%)	10 (45.5%)	
N3	20 (46.5%)	8 (38.1%)	12 (54.5%)	

IQR, interquartile range; EGFR-TKI, epidermal growth factor receptor tyrosine kinase inhibitor; LN, lymph nodes.

### Treatment feasibility

As **Table 2** demonstrated that the efficacy of neoadjuvant *EGFR*-TKI therapy was assessed by RECIST (version 1.1). PR was observed in 26 patients, and SD was observed in 16 patients. Only one patient refused to evaluation in our center after neoadjuvant treatment. The objective response rate (ORR) in this study was 61.9% (26/42). There was no CR radiologically. The distribution of tumor response was similar between T-S-Arm and T-Arm. After *EGFR*-TKI treatment, the rate of patients occurred radiologically CR in lymph nodes (N0) was higher in T-S-Arm (52.4% vs 27.3%, *P* = 0.148). Only one patient discontinued *EGFR*-TKI therapy for serious adverse event (interstitial pneumonia).

**Table 2:** Efficacy of patients receiving neoadjuvant *EGFR*-TKI plus complete resection or *EGFR*-TKI alone.

	Total (N=43)	T-S-Arm (N=21)	T-Arm (N=22)	*P* value
Tumor response				1.000
PR	26 (60.5%)	13 (61.9%)	13 (59.1%)	
SD	16 (37.2%)	8 (38.1%)	8 (36.4%)	
Missing	1 (2.3%)	0 (0%)	1 (4.5%)	
Clinical T stage after treatment				0.871
T1	24 (55.8%)	13 (61.9%)	11 (50.0%)	
T2	15 (34.9%)	7 (33.3%)	8 (36.4%)	
T3	1 (2.3%)	0 (0%)	1 (4.5%)	
T4	2 (4.7%)	1 (4.8%)	1 (4.5%)	
Missing	1 (2.3%)	0 (0%)	1 (4.5%)	
Pathological T stage after treatment				-
T1	11 (25.6%)	11 (52.4%)	0 (0%)	
T2	9 (20.9%)	9 (42.9%)	0 (0%)	
T3	0 (0%)	0 (0%)	0 (0%)	
T4	1 (2.3%)	1 (4.8%)	0 (0%)	
Missing	22 (51.2%)	0 (0%)	22 (100%)	
Pathological N stage after treatment				-
N0	11 (25.6%)	11 (52.4%)	0 (0%)	
N1	2 (4.7%)	2 (9.5%)	0 (0%)	
N2	8 (18.6%)	8 (38.1%)	0 (0%)	
Missing	22 (51.2%)	0 (0%)	22 (100%)	
LN response				0.148
Downstaging to N0	17 (39.5%)	11 (52.4%)	6 (27.3%)	
Downstaging to N1 or N2	9 (20.9%)	5 (23.8%)	4 (18.2%)	
Unchanged	16 (37.2%)	5 (23.8%)	11 (50.0%)	
Missing	1 (2.3%)	0 (0%)	1 (4.5%)	
Recurrence or progression				<0.001
Yes	31 (72.1%)	10 (47.6%)	21 (95.5%)	
No	12 (27.9%)	11 (52.4%)	1 (4.5%)	
Recurrence or progression position				<0.001
Local	19 (44.2%)	1 (4.8%)	18 (81.8%)	
Distance	12 (27.9%)	9 (42.9%)	3 (13.6%)	
None	12 (27.9%)	11 (52.4%)	1 (4.5%)	
Status				0.660
Alive	32 (74.4%)	15 (71.4%)	17 (77.3%)	
Dead	5 (11.6%)	3 (14.3%)	2 (9.1%)	
Censored	6 (14.0%)	3 (14.3%)	3 (13.6%)	

EGFR-TKI, epidermal growth factor receptor tyrosine kinase inhibitor; LN, lymph nodes; PR, partial response; SD, stable disease.

Patients in T-S-Arm all received neoadjuvant treatment followed by complete resection. There were 19 (19/21, 90.5%) patients receiving lobectomy, and 1 (1/21, 4.8%) receiving sleeve resection in T-S-Arm. There was one (1/21, 4.8%) patient receiving segmentectomy for poor pulmonary function. The median operation time was 2.25 hours (IQR, 2.00-3.00), and the median blood loss was 50.0 mL (IQR, 50.0-150.0) (**Table 3**). Perioperative complications included one (1/21, 4.8%) patient with pulmonary embolism (PE) and one (1/21, 4.8%) patient with pleural effusion. No surgical related death was observed within 90 days postoperatively.

**Table 3:** Surgical information and adjuvant therapy of patients in T-S-Arm.

	T-S-Arm (N=21)
Operative procedure	
Lobectomy	19 (90.5%)
Segmentectomy	1 (4.8%)
Sleeve resection	1 (4.8%)
Surgical approach	
VATS	14 (66.7%)
Open	7 (33.3%)
**Median operation time, h (IQR)**	2.25 [2.00, 3.00]
**Median blood loss, mL (IQR)**	50.0 [50.0, 150.0]
Perioperative complications	
Pulmonary embolism	1 (4.8%)
Pleural effusion	1 (4.8%)
None	19 (90.5%)
Adjuvant therapy	
*EGFR*-TKI	17 (81.0%)
Chemotherapy	4 (19.0%)

VATS, video-assisted thoracic surgery; IQR, interquartile range; EGFR-TKI, epidermal growth factor receptor tyrosine kinase inhibitor.

There were 17 (17/21, 81.0%) patients in T-S-Arm receiving *EGFR*-TKI for at least 2 years or until disease progression postoperatively. And four (4/21, 19.0%) patients received adjuvant chemotherapy every 3 weeks for 4 cycles. No patient received the third-generation *EGFR*-TKI as an adjuvant therapeutic regimen.

### Survival analysis

**Figure 2:**
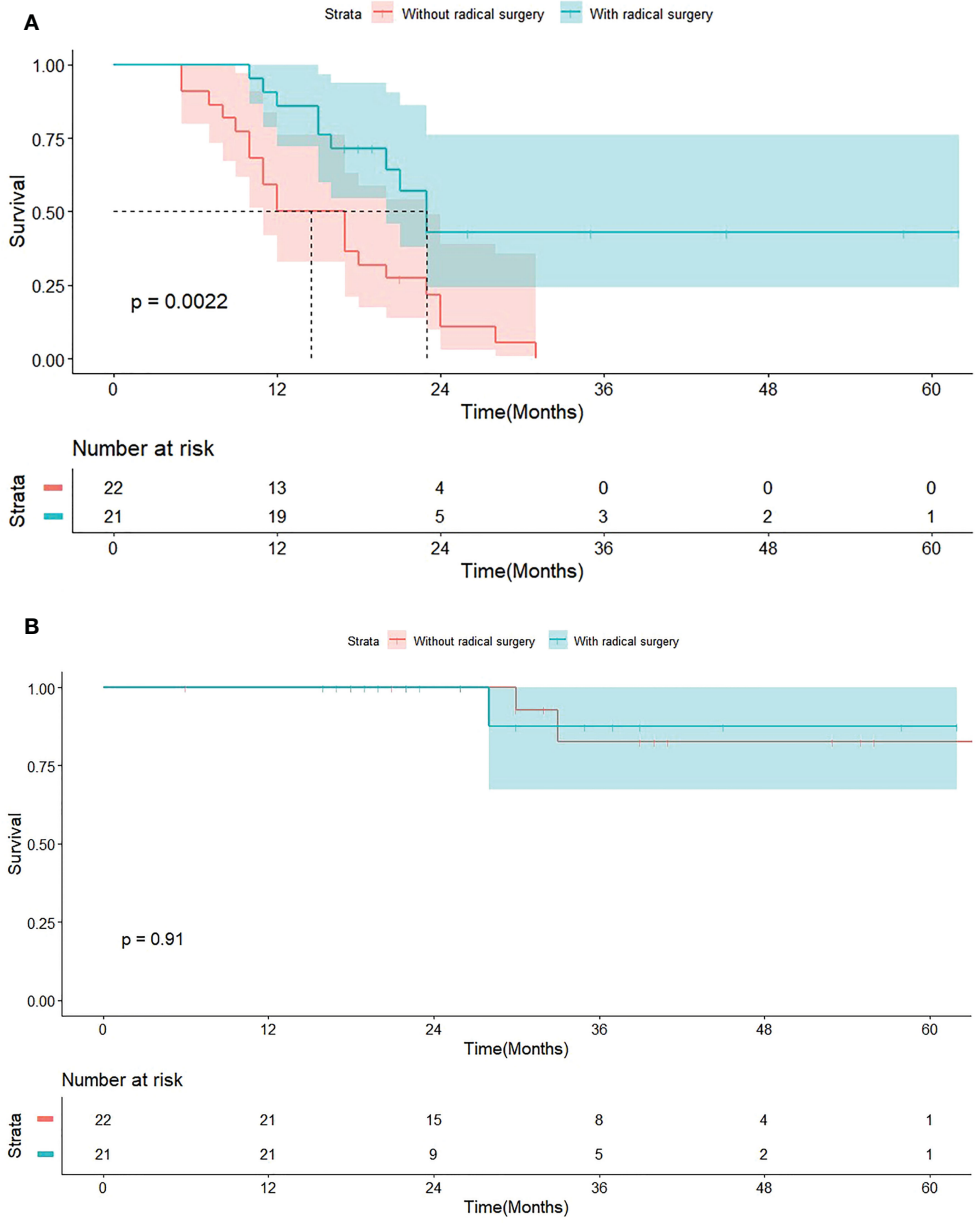
Survive curves for 43 patients with *EGFR*-TKI treatment. **(A)** Progression-free survival (PFS) grouped by patients with or without radical surgery. **(B)** Overall survival (OS) grouped by patients with or without radical surgery.

The median follow-up time was 28 months (IQR, 20-39). In T-S-Arm, there were 10 (10/21, 47.6%) patients occurring tumor recurrence, including one (1/10, 10%) local recurrence and nine (9/10, 90%) distant recurrence. As **Figure 2A** illustrated that the median PFS of patients in T-S-Arm was significantly better than those in T-Arm (23.0 months vs 14.5 months, *P* = 0.002). Improvement of PFS at 1-year (85.7% vs 50.0%, *P* = 0.021) was also observed in T-S-Arm. PFS at 2-year (42.9% vs 10.9%, *P* = 0.103) was similar between T-S-Arm and T-Arm. As of the final follow-up date, 32 (32/43, 74.4%) patients survived, 6 (6/43, 14%) were lost to follow-up after recurrence or progression and 5 (5/43, 11.6%) died of distant metastasis (3 in T-S-Arm and 2 in T-Arm). The median OS did not reach in both groups (**Figure 2B**).

**Figure 3:**
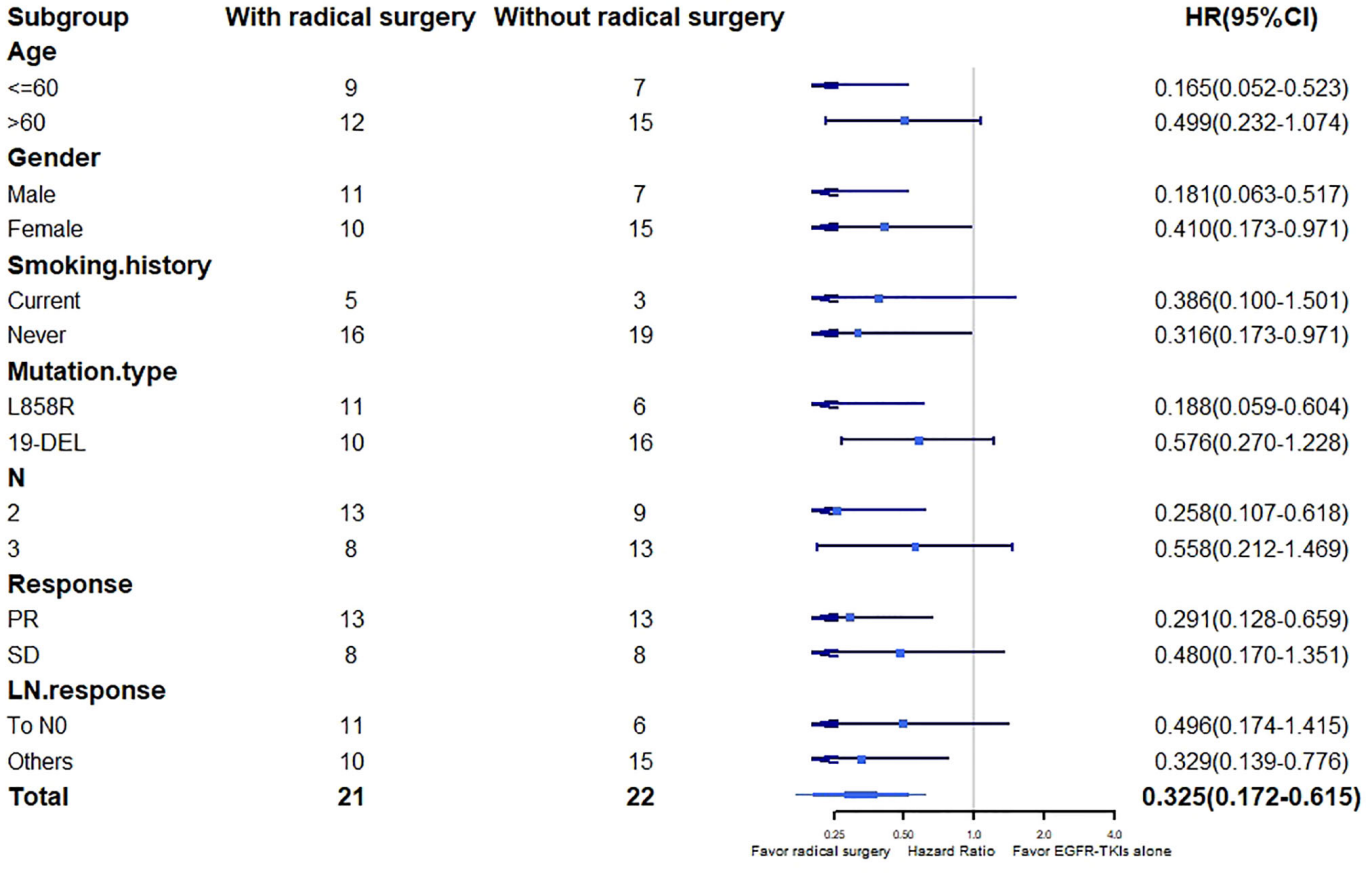
PFS in patient’s subgroups. PFS, progression-free survival; *EGFR*-TKI, epidermal growth factor receptor tyrosine kinase inhibitor; PR, partial response; SD, stable disease; LN, lymph nodes; HR, hazard ratio; CI, confidence interval.

Multivariate Cox regression analysis showed that radical surgery (HR, 0.406; 95% CI, 0.207-0.793; *P* = 0.027) was the only independent predictive factor for disease progression (**Table 4**). In the stratified analysis, PFS favored radical surgery in younger patients (HR, 0.165; 95% CI, 0.052-0.523; *P* = 0.010), non-smokers (HR, 0.316; 95% CI, 0.137-0.971; *P* = 0.010), harboring L858R mutation (HR, 0.188; 95% CI, 0.059-0.604; *P* = 0.019), with stage N2 disease (HR, 0.258; 95% CI, 0.107-0.618; *P* = 0.011), radiological PR (HR, 0.291; 95% CI, 0.128-0.659; *P* = 0.013) and without lymph node CR (HR, 0.329; 95% CI, 0.139-0.776; *P* = 0.033). There was no significant difference in other subgroups (**Figure 3**).

**Table 4:** Univariate and multivariate Cox regression analysis for PFS in patients with receiving *EGFR*-TKIs.

	Univariable		Multivariable	
Variables	HR (95% CI)	*P* Value	HR (95% CI)	*P* Value
Age		0.403		
<60	1.000			
≥60	1.380 (0.732-2.599)			
Gender		0.686		
Female	1.000			
Male	1.162 (0.631-2.139)			
Smoking history		0.547		
Never	1.000			
Current or ever	1.318 (0.621-2.798)			
Mutation type		0.060		0.293
L858R	1.000		1.000	
19-del	2.125 (1.100-4.103)		1.554 (0.780-3.097)	
**Target lesions**	0.996 (0.982-1.010)	0.637		
Stage N		0.235		
II	1.000			
III	1.555 (0.844-2.867)			
Tumor response		0.802		
PR				
SD	0.909 (0.486-1.700)			
LN response		0.083		0.175
To N0	1.000		1.000	
To N1 or unchanged	1.977 (1.035-3.776)		1.721 (0.891-3.323)	
Therapy		0.004		0.027
Without radical surgery	1.000		1.000	
With radical surgery	0.325 (0.172-0.615)		0.406 (0.207-0.793)	

LN, lymph nodes; HR, hazard ratio; CI, confidence interval; PR, partial response; SD, stable disease; EGFR-TKI, epidermal growth factor receptor tyrosine kinase inhibitor.

## Discussion

The radical surgery remains uncertain in stage IIIB NSCLC patients harboring *EGFR* mutations occurring downstaging after neoadjuvant *EGFR*-TKI therapy. Recent studies have reported a small sample of *EGFR*m NSCLC patients in stage IIIB receiving salvage surgery following *EGFR*-TKI treatment and demonstrated the feasibility of neoadjuvant *EGFR*-TKI therapy for *EGFR*m NSCLC in stage IIIB (12, 13). The results suggested that neoadjuvant *EGFR*-TKI therapy in combination with radical surgery could provide significantly better median PFS with *EGFR*m LUAD patients in stage IIIB compared with these patients receiving *EGFR*-TKI alone (23.0 months vs 14.5 months, *P* = 0.002).

The randomized phase II study (EMERGING-CTONG1103) reported the median PFS of 21.5 months (95% CI, 16.7-26.3) of NSCLC patients with stage IIIA-N2 disease receiving neoadjuvant erlotinib therapy combined with radical surgery and the ORR was 54.1% (20/37) (14). The open label phase III study (WJTOG3405) showed that median PFS of *EGFR*m NSCLC patients in stage IIIB (6th edition TNM classification, including T3N3 and T4N3 disease) receiving gefitinib was 13.7 months (95% CI, 7.2-20.5), and the ORR was 62.1% (36/58) (15). The median PFS and ORR of patients in T-Arm were consistent with those in WJTOG3405. However, the median PFS and ORR of patients in T-S-Arm were slightly higher than those in EMERGING-CTONG1103. One possible reason is that we excluded patients with primary resistance to *EGFR*-TKIs who had worse PFS, while there were 3 patients with PD in EMERGING-CTONG1103. Another reason is that the longer period of neoadjuvant *EGFR*-TKI might provide enough time for tumor to decrease. In our study, the duration of neoadjuvant *EGFR*-TKI varied 1 to 6 months, while patients in EMERGING-CTONG1103 received preoperative *EGFR*-TKI for 42 days.

The preferred treatment for NSCLC patients with N3 disease is systemic therapy instead of surgery. In 2018, Ning et al. reported 2 *EGFR*m NSCLC patients in stage IIIB receiving complete resection after *EGFR*-TKI treatment. Both of them were in stage N2 and downstaged to N0 preoperatively (16). In 2021, Li et al. collected 91 NSCLC patients with unresectable disease before receiving *EGFR*-TKI treatment, and 18 of them downstaged and received salvage resection after *EGFR*-TKI treatment. There were 3 patients with N3 disease undergoing surgery, achieving a mean PFS of 15.8 months (range, 8.5-26 months) (13). In our study, patients with stage N3 disease in T-S-Arm were carefully assessed as downstaging to make sure that all of them could receive radical surgery. However, subgroup analysis showed that there was no significant difference between T-S-Arm and T-Arm in patients with N3 disease. Therefore, radical surgery might be unsuitable for patients with N3 disease. It was also reported that surgery might improve long-term survival compared with chemoradiation in patients with N3 disease (17). Unfortunately, it could not be demonstrated in our study for the follow-up time was too short. Large-scale clinical trial and longer period of follow-up are needed to confirm the feasibility of treatment strategies aiming at complete resection in *EGFR*m NSCLC patients with N3 disease.

In previous studies, patients with 19-DEL were generally more sensitive to *EGFR*-TKIs than those with L858R mutation (18, 19). Kuan et al. demonstrated that *EGFR*-TKIs could improve PFS of patients with 19-DEL (HR, 0.27; 95% CI, 0.21-0.35) and L858R mutation (HR, 0.45; 95% CI, 0.35-0.58), but there was no benefit for OS of patients with L858R mutation (20). In EMERGING-CTONG1103, there was no significant difference in DFS between patients with L858R mutation and 19-DEL receiving neoadjuvant *EGFR*-TKI therapy combined with radical surgery (21.9 months vs 21.7 months) (14), and this conclusion was confirmed in this study. However, PFS of patients with L858R mutation receiving radical surgery was significantly improved compared with *EGFR*-TKI alone (HR, 0.188; 95% CI, 0.059-0.604), indicating that patients with L858R mutation could benefit more from radical surgery than those with 19-DEL. In previous studies, it was observed that T790M, which was sensitive to the third-generation of *EGFR*-TKIs, was more frequent in patients with resistance to *EGFR*-TKIs harboring 19-DEL than those harboring L858R mutation (21). Therefore, there are more options of second-line therapies for patients with 19-DEL than those with L858R mutation. In this study, we found that radical surgery could provide the alternative for patients with L858R mutation. Future clinical trials should take 19-DEL and L858R mutation as distinct factors and individualize treatment plans.

Lymph nodes clearance after neoadjuvant therapy was proved to be a prognostic of survival for NSCLC patients with stage III disease (22, 23). However, Andrews et al. showed that there was no significant difference in survival between patients with persistent N2 disease and with mediastinal downstaging undergoing complete resection (24), which was also observed in this study. For pre-induction N2 disease, radical surgery is recommended after *EGFR*-TKI therapy even if there is persistent N2 disease preoperatively.

Our study had limitations: (I) As this study was retrospective, selection bias was inevitable and could affect the results of this study. Randomized controlled clinical trials were needed for further confirmation. (II) The sample size was too small to define the difference of patients in stage N3 with downstaging after *EGFR*-TKI treatment between T-S-Arm and T-Arm, for radical surgery was not the routine treatment for these patients according to clinical guidelines. (III) The follow-up duration was relatively short. (IV) The information of survival status and therapeutic regimens after disease progression or recurrence was missing for some patients during follow-up. Thus, the OS was not discussed in this study profoundly. Further studies would extend the follow-up duration and analyze the treatment strategies for post-recurrence. (IV) The clinical N stage before treatment was evaluated according to the SUV in PET/CT, for the information of pathological lymph nodes involvement before treatment of a part of patients was missing. (V) Information about adverse events in grade 1 to 2 was missing for the majority of patients. However, it is highlighted that *EGFR*-TKIs were well-tolerated and the treatment protocols were well-established according to previous clinical trials.

In conclusion, neoadjuvant *EGFR*-TKI in combination to radical surgery could improve the prognosis of *EGFR*m LUAD patients in stage IIIB, especially for patients with N2 disease and harboring L858R mutation. Radical surgery should be carefully selected for patients with N3 disease after *EGFR*-TKI treatment.

## Data availability statement

The original contributions presented in the study are included in the article/supplementary materials. Further inquiries can be directed to the corresponding authors.

## Author contributions

YX and DB conceived and designed the analysis. Data collection was performed by YX and ZH. Analysis and interpretation of the data were supported by YX, DB, HY, and JH. PZ, WH, and HL revised the manuscript critically for important intellectual content. All authors participated in manuscript writing and approved the final manuscript.
